# Proceedings of the American Proctological Society

**Published:** 1910-10

**Authors:** 


### PROCEEDINGS OF THE AMERICAN PROCTOLOGICAL SOCIETY.

*Continued.*

### "ATONY OF THE RECTUM."

By William M. Beach, M. D., of Pittsburgh, Pa.

Dr. Beach stated that atony or sluggishness of the rectum signifies the inability to expel its contents by reason of impaired musculature, ligimentation or innervation, and further that the musculature in the rectum proper, or that portion above the plane of the levator ani is entirely involuntary whose inertia must therefore be due to some inherent factor.

On the contrary, the anal canal, which is made up for the most part of the voluntary fiber has most to do with the expulsive act, the normal function of which depends chiefly upon the muscular automaton tha is intact, proper innervation and psychic influence.

The physiologic rectum depends upon (1) an unobstructed canal (2) firm ligaments, and (3) a well-developed rectal sense residing in the anal canal. Factors contributing to atony are (a) Traumatism to the perineal body, (b) disease in the anal canal, (c) Enteroptosis secondary to general systemic conditions or local anatomic anomalies, (d) the abuse of injections and drastic catharsis, (e) disease in adjacent organs, as prolapsed uterus, adhesions, neoplasms, appendicitis, prostatitis, corculatory disturbance as engorged portal vessels and primary gastric diseases, (f) atony may be the sequel to luesis or senility. The treatment is that of constipation being guided by the cause. Alterative, dietetic and mechanical agencies are to be invoked.

### "VILLOUS TUMOR OF THE RECTUM."

By T. Chittenden Hill, M. D., of Boston, Mass.

The author stated that a villous tumor of the rectum is very uncommon and but few cases have been recorded in current literature. B. Merrill Ricketts reported a case before this society in 1907 and states that but "Sixty-two cases have been reported, nine of which have been by six American authors." Since then I have been able to find but one case reported by Vautrin—(L'Review de la Gynecologia.) His article is the most accurate and painstaking observation to be found on the subject.

It is rather difficult to arrive at any conclusion as to their relative frequency by studying the reported cases or by searching hospital reports, as these border-line tumors are generally very loosely classified. Probably the most acurate data at our disposal may be had from St. Marks Rectal Hospital, London, in which twenty-five villous tumors are tabulated among 42343 patients with rectal ailments.

The chief point of interest about these tumors is that a certain percentage of them show a marked tendency to undergo malignant degeneration. From the histories of the 13 cases cited by Ricketts, including one of his own, we learn that three recurred and three did not. Those with a broad base, later became malignant, while those with a pedicle did not. . Of the other seven cases no mention was made as to the final outcome.

Goodsall and Miles have had 12 cases--8 in men and 4 in women, of which number two ultimately became carconomatous.

From careful study of these cases and several others the author believes that if there is a distinct pedicle without infiltration of the adjacent mucous membrane, tumors of this type are generally benign and if completely removed by ligation, or otherwise, there is but little likelihood of their recurring. On the other hand, if the base is broad, whether there be induration or not, a total extirpation of the rectum should be advised.

Another point of some interest borne out by a study of these cases is that the longer the condition has existed the less likely is it that the growth will prove malignant. The case now reported seems to bear out this statement.

Mrs. M., forty years of age, was referred by Doctor J. H. Vaugn of Everette, Mass., Jan. 5, 1907. She was well-nourished, weight about normal, but anemic, with shallow complexion. Had had indigestion for years but in other respects was in good health. For the past six years had noticed small rectal hemorrhages. During the year previous the hemorrhages had become more profuse and the mass was always protruded at the anus during defecation and even after slight exertion when walking.

She had to go to the toilet several times during the day and to get up two or three times at night, when she would pass one-half cupful of blood-tained micous; also considerable mucous would at times escape with flatus. For two months, tenesmus had been present nerly all the time. She did not complain of anal or sacral pain.

Rectal examination. Sphincters, epri-anal skin and nal canal, were perfectly normal. In the rectum was felt a slippery growth with a band-like pedicle one inch wide by one-half inch thick, attached obliquely with the long axis of the rectum. By careful manipulation the writer was able to bring outside the anal orifice, a lobulated cauliflower like mast, the size and shape of a large English walnut, from which there was a gentle oozing of blood while it was held outside by the sphincters.

Operation January 8th., 1907. The sphincters were stretched after infiltration with one quarter of 1% cocaine solution and the mass drawn down with the finger and the pedicle infiltrated and clamped about half an inch from the margin of the tumor.

The Pedicle was then transfixed on the proximal side of the clamp and ligated with pagenstechers No. 5 in three sections and the pedicle cut away on the distal side. An ounce of bloody muscous escaped from the anus during the dilation.

The operation was easily performed and with but little discomfort to the patient under local anesthesia.

Over three years have now elapsed since the case was operated upon and as yet there is no sign of recurrence.

The report of Dr. Louis Hoag upon speciment, Jan. 8, 1907, wa sas follows:- "Pedunculated cauliflower tumor of flattened spheroidal form of pale brownish red color and 4x3½ cm. in size.

'Surface quite regularly broken by deep narrow pits and furrows between and among hundreds of small hemispherical ovoid and psindle-shaped lobules ranging from 1 to 3 mm. in diameter. Such are soft, juicy but not necrotic and of uniform pale brownish red color. Surface always smooth and glistening. Irregularly distributed are deeper clefts outlining pyramidal divisions of the tumor, each bearing upon its base, which is directed outward, a number of the lobules just described.

'Toward the periphery of the cross section of the tumor the lobules are of uniform-soft consistency and of uniform pale-brown red color. Centrally the pale pedicles, which are about 4mm. in diameter, enter the tumor at a sort of hilus and its white fibrous tissue bearing numerous small blood-vessels spreads out to be finally lost in the similiar tissue of the apices of the various pyramidal divisions of the tumor."

(TO BE CONTINUED)

